# *Albizia julibrissin* Ameliorates Memory Loss Induced by Insomnia in* Drosophila*

**DOI:** 10.1155/2019/7395962

**Published:** 2019-04-01

**Authors:** Jui-Shu Chang, Hsin-Ping Liu, Jack Cheng, Chao-Jung Chen, Su-Lun Hwang, Chi-Chuan Tseng, Lee-Fen Hsu, Wei-Yong Lin

**Affiliations:** ^1^Graduate Institute of Integrated Medicine, China Medical University, Taichung 40402, Taiwan; ^2^Graduate Institute of Acupuncture Science, China Medical University, Taichung 40402, Taiwan; ^3^Department of Bioinformatics and Medical Engineering, Asia University, Taichung 41354, Taiwan; ^4^Department of Medical Research, China Medical University Hospital, Taichung 40402, Taiwan; ^5^Department of Nursing, Chang Gung University of Science and Technology, Chiayi County 61363, Taiwan; ^6^Division of Pulmonary and Critical Care Medicine, Chang Gung Memorial Hospital, Chiayi County 61363, Taiwan; ^7^Division of Chinese Medicine, Chang Gung Memorial Hospital, Chiayi County 61363, Taiwan; ^8^Department of Respiratory Care, Chang Gung University of Science and Technology, Chiayi County 61363, Taiwan; ^9^Division of Neurosurgery, Department of Surgery, Chang Gung Memorial Hospital, Chiayi County 61363, Taiwan; ^10^Brain Diseases Research Center, China Medical University, Taichung 40402, Taiwan

## Abstract

In clinical practice in Taiwan,* Albizia julibrissin* is the most prescribed Chinese herbal medicine for insomnia. Short-term insomnia and hypnotic use both attenuate cognitive functions, especially learning memory. In previous studies,* A. julibrissin* exhibits sedative activity, antidepressant-like effects, and protection of learning and memory against amnesia. However, whether* A. julibrissin* ameliorates memory loss caused by short-term sleep deprivation is not clear. We utilized the sleep-deprived* Drosophila* model and olfactory associative learning-memory assay to test the effects of* A. julibrissin* on sleep-deprivation induced memory loss. We found that* A. julibrissin* ameliorated 3-hour memory but not 1-hour memory or instant learning. The findings might be applied to an anticipated short-term sleep disturbance.

## 1. Introduction

Insomnia dominates the category of sleep disorder and continues to be a rising public problem in recent years, prevalence ranging from 15% to 50% [1]. Although cognitive behavioral therapy for insomnia (CBT-i) is gradually emphasized, as suggested in the 2017 European guideline for the diagnosis and treatment of insomnia as the recommended first-line treatment for chronic insomnia, pharmacological intervention is still indispensable when CBT-i is not available or is not sufficient [2]. Moreover, pharmacological treatment such as benzodiazepines and benzodiazepine receptor agonists are recommended for short-term insomnia [2], with careful management to minimize their drug dependence, including tolerance, escalation of dosage, and a withdrawal syndrome [3]. In practice, the hypnotic use is high (16.2%) in patients visiting their general practitioner [4]. However, the increased risk for dementia and fractures was associated with the use of hypnotics for insomnia as revealed in a meta-analysis [5].

Besides, sleep disturbance itself causes cognitive deficits. Interestingly, different types of sleep problems lead to distinct profiles of the disturbed domain of cognition [6]. Since short-term sleep deprivation (i.e., 1-2 nights) but not long-term insomnia causes deficits in attention and memory [6], an investigation into assisting with memory problems due to short-term sleep disruption is necessary.

In clinical practice of Taiwan, 87% of patients with sleep disorder take “Western” medicine, while 12% of them take Chinese herbal medicine [7].* Albizia julibrissin* is one of the most common individual Chinese herbs consumed for insomnia and occupies 10% of the prescription, included as a component in the compound formula (Fu-Fang, regimen, or remedy) in Taiwan [8]. In traditional Chinese medicine (TCM) practice,* A. julibrissin* is also prescribed for liver depression and qi stagnation [9]. In preclinical studies,* A. julibrissin* exhibits sedative activity [10, 11], anxiolytic effects [12, 13], and antidepressant-like effects [14]. Moreover,* A. julibrissin* treatment in mice shortened sleeping onset time and prolonged sleep duration time [11, 15] and protected learning and memory against amnesia induced by scopolamine [16]. Due to these cues, we hypothesized that* A. julibrissin* ameliorates memory loss caused by short-term insomnia or sleep deprivation.

In the field of sleep and circadian rhythms, discoveries in* Drosophila melanogaster* model (fruit flies) have led the fundamental breakthroughs and are found to be conserved in the mammal, including identification of first core clock gene and mechanism for photic resetting [17]. As a tool to study learning memory, olfactory learning of* Drosophila* shares the same characteristics of Pavlovian learning with other animals, including acquisition, extinction, conditioned excitation, and conditioned inhibition [18]. Furthermore, Drosophila shares similar molecular pathway with the mammal for memory storage, such as cAMP-PKA-CREB [19].

The recommended dosage of the extracted* A. julibrissin* is 3~6 g each time for an adult according to the Taiwan Herbal Pharmacopoeia [20], and the usual prescription of three times per day leads to a daily dose of 9~18 g, which is equivalent to 4.5~9 mg/ml approximately. In this study, we evaluated the efficacy of* A. julibrissin* treatment, with two doses of 4.5 and 18 mg/ml, on the learning and memory of sleep-deprived* Drosophila* model.

## 2. Methods

### 2.1. Maintenance of* Drosophila* and Sleep Deprivation

Canton-S is obtained from Bloomington Drosophila Stock Center and maintained with cornmeal-sucrose-yeast culture medium under 12h day-night shift at 25°C. For evaluating the impact of disturbance on learning and memory, flies at the age of 5 to 7 day after emergence were used. For assessing the effect of protection of* A. julibrissin* treatment on learning and memory disturbed by sleep deprivation, flies were treated with* A. julibrissin* of different concentrations, i.e., 0, 4.5, and 18 mg/ml, for 5 days since emergence and subjected to sleep deprivation at the night of the 5^th^ day for 12h, and then subjected to T-maze for olfactory associative learning at the 6^th^ day. For sleep deprivation,* Drosophila* culture bottles were fixed on the orbital shaker (VWR VX2500) at 850 rpm during ZT 13-24. The culture bottles of the control were placed at the same space.

### 2.2. Preparation of Albizia julibrissin

Fresh flowers of* A. julibrissin* (6 kg) were boiled with water (6 liters) for 1hr, filtered out precipitant, water bathed and concentrated to 150 ml, and freeze-dried to 90 g powder. The powder was added to and mixed thoroughly with a food processor in the* Drosophila* culture medium before solidification. The final concentration of* A. julibrissin* powder in culture medium was 4.5 and 18 mg/ml.

### 2.3. Profiling of Extract by LC-ESI-Q-TOF

For the quality control of* A. julibrissin* extracts, spectrometry was undertaken. An ultra-high performance liquid chromatography (UHPLC) system (Ultimate 3000; Dionex, Germany) equipped with a C18 reversed-phase column (2.1 × 150 mm, 3 *μ*m, T3; Waters, Milford, MA, USA) was coupled with a hybrid Q-TOF mass spectrometer (maXis impact, Bruker Daltonics, Bremen, Germany) with an orthogonal electrospray ionization (ESI) source. A volume of 2 *μ*L of sample was injected. The LC flow rate was 0.20 mL/min. At the beginning of elution, 100% solvent A (0.1% formic acid) was used, and then the solvent B (acetonitrile with 0.1% formic acid) was increased to 60% during a span of 17 min and finally to 100% over a period of 0.1 min after which this percentage composition was held for 1.9 min. After 0.1 min, solvent B was reduced back down to 0% and held at this percentage for 3.9 min. The mass spectrometer was operated in either positive or negative ion mode using the m/z range 50~1000 at 1 Hz. The capillary voltage of the ion source and negative was set at +4200 V for the positive mode. The nebulizer gas flow was 1 bar and drying gas flow was 8 L/min. The drying temperature was set at 200°C. The HPLC fingerprint for quality control of* A. julibrissin* powder is shown in Supplementary Figure 1.

### 2.4. Olfactory Associative Learning-Memory Assay (T-Maze)

**Figure 1:**
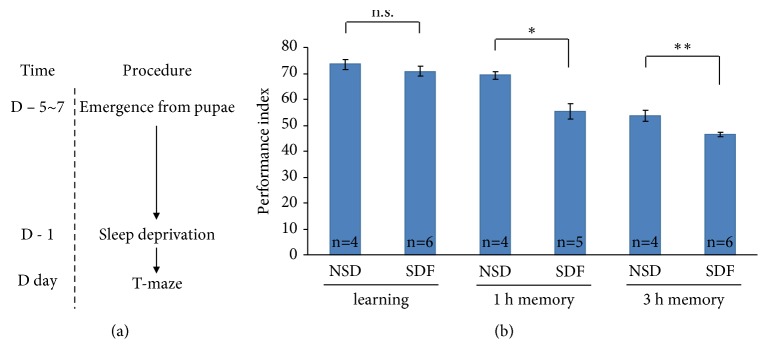
*Sleep deprivation impaired 1-h and 3-h memory of Drosophila*. (a) The experiment program. (b) The performance index of instant learning, 1 h memory, and 3 h memory of sleep-deprived* Drosophila*. “SDF” denotes sleep-deprived flies, while “NSD” denotes non-sleep-deprived control. *∗* and *∗∗* denote p < 0.05 and 0.01 compared to NSD, respectively. Error bar stands for the standard error of the mean.

**Figure 2:**
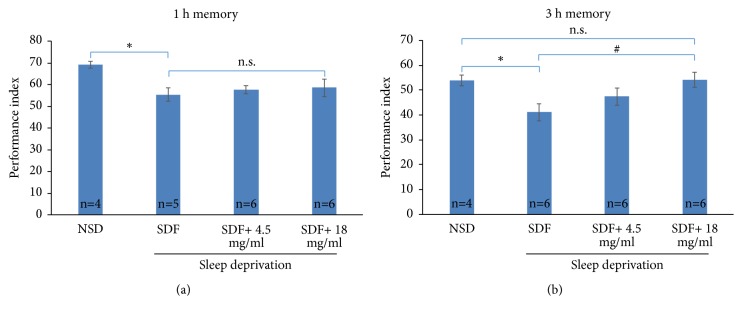
*A. julibrissin ameliorated sleep-deprivation induced 3-h memory impairment*. (a) The performance index showing 1 h memory of* Drosophila*. All doses of* A. julibrissin* did not rescue the impairment induced by sleep deprivation. (b) The performance index showing 3 h memory of* Drosophila*. High dose of* A. julibrissin* rescued the impairment induced by sleep deprivation. “SDF” denotes sleep-deprived flies, while “NSD” denotes non-sleep-deprived control. Doses of* A. julibrissin* are in mg/ml. # denotes p < 0.05 compared to SDF by one-way ANOVA and post hoc Dunnett's multiple comparisons test. *∗* denotes p < 0.05 compared to NSD by Student's T-test. Error bar stands for the standard error of the mean.

**Figure 3:**
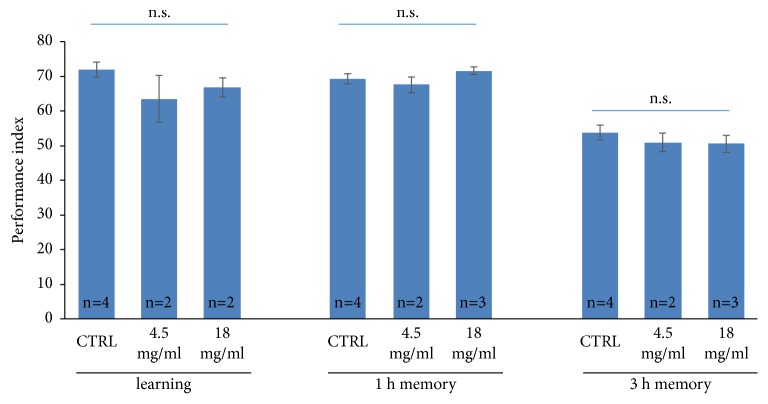
*A. julibrissin did not alter learning memory of Drosophila without sleep deprivation*. The performance index of instant learning, 1 h memory, and 3 h memory of* A. julibrissin* treated* Drosophila*. “CTRL” denotes untreated control. Doses of* A. julibrissin* are in mg/ml. Error bar stands for the standard error of the mean.

The training session was performed according to a previous study [21]. Briefly, approximately 100 flies were exposed sequentially to two odors, either 18 *μ*M of 3-octanol (OCT) or 13 *μ*M of 4-methylcyclohexanol (MCH). One biological replicate contains two bottles of flies for these two odors, respectively. Flies exposed to the first odor were simultaneously supplied with 80~90 V electric shocks and then received a second odor control group without electric shocks. Learning ability was determined immediately after training, while 1-h or 3-h memory was determined after 1 or 3 h after training. To perform the test, the trained flies were loaded into the choice point of a T-maze in which they were exposed simultaneously to OCT and MCH. A performance index (PI) was calculated to represent the conditioned odor avoidance. The biological replicates are indicated in Figures 1(a), 2(a), 2(b), and 3.

### 2.5. Statistics

To evaluate the effect of disturbance on learning and memory, the significance of the difference between performance indexes of flies with and without sleep deprivation was calculated with Student's T-test. To evaluate the effect of protection of* A. julibrissin* treatment on learning and memory disturbed by sleep deprivation, one-way ANOVA and post hoc Dunnett's multiple comparisons test was applied. The p value < 0.05 was considered significant.

## 3. Results

### 3.1. Sleep Deprivation Impaired 1-h and 3-h Memory of* Drosophila*

To test our hypothesis that* A. julibrissin* ameliorates memory loss caused by short-term insomnia or sleep deprivation by using* Drosophila* model, as an internal control, we have had to show that sleep deprivation impairs memory and to determine which type of memory is influenced in advance. The control experiment program is showed in Figure 1(a). Flies of the age of 5 ~ 7 days after emergence, without* A. julibrissin* treatment, were sleep deprived for one night (the SDF group) or not sleep deprived (the NSD group) at the day just before memory test and were subjected to olfactory associative learning (T-maze) assay to evaluate instant learning, 1-h memory, and 3-h memory. As shown in Figure 1(b), sleep deprivation impaired 1-h and 3-h memory, but not instant learning of* Drosophila*.

### 3.2. *A. julibrissin* Pretreatment Ameliorated Sleep-Deprivation Induced 3-h Memory Impairment

Next, we tested whether pretreatment of* A. julibrissin* protects memory against sleep deprivation. Emerged flies were divided into three groups of* A. julibrissin* treatment with different concentrations, i.e., 0, 4.5, and 18 mg/ml, for five days and were subjected to sleep deprivation for one night. At the next day after sleep deprivation, flies were subjected to olfactory associative learning-memory assay (T-maze) for 1-h memory and 3-h memory tests. As shown in Figure 2(a),* A. julibrissin* treatment did not improve 1-h memory. However,* A. julibrissin* of high concentration (18mg/ml) protected 3-h memory against sleep deprivation (Figure 2(b) and Tables 1 and 2).

### 3.3. *A. julibrissin* Did Not Alter Learning and Memory of* Drosophila* without Sleep Deprivation

To rule out that the effect of pretreatment of* A. julibrissin* is a confounding result from its ability to elevate memory capacity rather than its protection against sleep deprivation, we tested the memory of flies with* A. julibrissin* treatment but without sleep deprivation. Emerged flies were divided into three groups of* A. julibrissin* treatment with different concentrations, i.e., 0, 4.5, and 18 mg/ml, for five days and were subjected to olfactory associative learning-memory assay (T-maze) for instant learning, 1-h memory, and 3-h memory tests. As shown in Figure 3, instant learning, 1-h memory, and 3-h memory were not significantly altered with* A. julibrissin* treatment. Therefore, we confirmed that* A. julibrissin* pretreatment ameliorates 3-h memory impairment by sleep deprivation, but not elevating memory capacity.

## 4. Discussion

In this study, we utilized* Drosophila* as the model of memory impairment by sleep disruption and justified our hypothesis that* A. julibrissin* ameliorates sleep-deprivation induced memory impairment. Our data showed that sleep deprivation impairs 1-h and 3-h memory but not the instant learning ability of* Drosophila*, which is consistent with the previous study [22]. With* A. julibrissin* treatment, only 3-h memory but not 1-h memory of sleep-deprived* Drosophila* was rescued. To the best of our knowledge, this is the first demonstration that* A. julibrissin* treatment rescues memory loss impaired by insomnia in an animal model.

The limitations of this study include that (1) only young subjects (5 days after emergence) were tested, and the findings cannot be extended directly to elderly subjects, who are more vulnerable to sleep deprivation [23]. (2) Only short-term sleep disturbance (one night) was tested. However, unlike short-term insomnia, the impact of chronic insomnia on memory is not significant [6]. Therefore, we omitted the test of chronic insomnia in this study. (3) We used the mixture of water extractables rather than a specific compound of* A. julibrissin*. Further studies to identify the most effective compound are essential for therapeutic development. (4) We did not perform cotreatment with other hypnotics, such as benzodiazepines, to confirm whether hypnotics interferes the protection to memory against sleep deprivation by* A. julibrissin*. (5) The treatment was given before sleep deprivation, which may imply the clinical application to be preventive, but not curative.

Phytochemical studies revealed different classes of secondary metabolites in genus* Albizia*, like triterpene saponins, flavonoids, and alkaloids [24]. Since flavonoids and alkaloids dissolve poorly in water, we suggest saponins from* A. julibrissin* to be the bioactive component for the effect against memory loss due to sleep disruption. However, this report does not rule out the possibility that flavonoids or alkaloids from* A. julibrissin* to be effective in the same scenario. Since the discovery of the first saponin julibroside J1 at 1996 [25], many julibrosides, including julibroside J1, JC1, J2, J3, J4, J5, J8, J9, J14, J15, J16, J20, J22, J25, J28, J29, J30, J31, and J35, and their isomers have been identified in* A. julibrissin* [26]. Among these saponins, some phytomedical characteristics have been determined, i.e., cytotoxicity for J1 to J3 [27] and J21 [28], antiangiogenicity for J8 [29], antitumor activity for J28 [30], J29, J30, and J31 [31], and anxiolytic-like effects for JC1 [13]. JC1 seems to contribute to the anti-insomnia effect of* A. julibrissin*, but which saponin(s) dominate the effect against memory loss due to sleep deprivation requires further investigation.

Although we did not study the underlying mechanism, previous studies of* A. julibrissin* have identified its effects on neural transmission. Aqueous extract of* A. julibrissin* increases 5-HT1A receptors in serotonergic neurons in the frontal cortex and hippocampus of rat brain [32]. Moreover,* A. julibrissin* shows antidepressant effects possibly through antagonizing the neuron apoptosis of hippocampus [33]. Furthermore,* A. julibrissin* prevents apoptosis in neurons from oxidative stress [34]. These studies together suggest that* A. julibrissin* protects neuron from oxidative stress, which may be induced by short-term insomnia [35].

Despite these limitations, this study breaks Chinese alternative medicine (CAM) into a specific herb, whereas normally CAM contains grouped herbs into one treatment [36]. Although the compound formula (Fu-Fang, regimen, or remedy) in CAM is well established in Chinese medicine tradition, the decomposition of the compound formula into a specific herb or even into a bioactive molecule may modernize CAM as a resource of drug development.

## 5. Conclusion

We found that* A. julibrissin* treatment ameliorated memory loss induced by sleep deprivation in* Drosophila* model. The findings in this study might be applied to an anticipated short-term sleep disturbance, such as a temporary night shift, stay up late for an exam, or jet lag. In the future study, we will focus on (1) side effects of long-term treatment, (2) using older subjects, (3) extracted compounds, and (4) comparison and cotreatment with hypnotic for* A. julibrissin* on insomnia. In conclusion, this is an innovative study that may provide a new pharmacological treatment to aid in protecting memory loss in individuals with short-term sleep loss.

## Figures and Tables

**Table 1:** One-way ANOVA and Dunnett's multiple comparisons test on the performance index of 3 h memory amelioration by *A. julibrissin* treatment.

One-way ANOVA		
F	3.801	
R square	0.3364	
P value	0.0462	
P value summary	*∗*	

Dunnett's multiple comparisons test		

Comparison	SDF vs. SDF+ 4.5 mg/ml	SDF vs. SDF+ 18 mg/ml
Mean Diff.	-6.341	-13.03
95.00% CI of diff.	-17.86 to 5.183	-24.55 to -1.501
Adjusted P Value	0.3284	0.027
P value summary	ns	*∗*

**Table 2:** *Student's T-test on the performance index of 3 h memory comparing SDF to NSD and SDF with A. julibrissin treatment to NSD.* “SDF” denotes sleep-deprived flies, while “NSD” denotes non-sleep-deprived control. Doses of *A. julibrissin* are in mg/ml.

Unpaired two-tailed T-test		
Comparison	NSD vs. SDF	NSD vs. SDF+ 18 mg/ml
t, df	t=2.728 df=8	t=0.07479 df=8
R squared (eta squared)	0.4819	0.0006986
Mean Diff.	-12.71 ± 4.661	0.3107 ± 4.155
95.00% CI of diff.	-23.46 to -1.966	-9.27 to 9.892
P value	0.0259	0.9422
P value summary	*∗*	ns

## Data Availability

The data used to support the findings of this study are available from the corresponding author upon request.

## Abbreviations

SDF: Sleep-deprived flies
NSD: Non-sleep-deprived flies
*A. julibrissin:*: * Albizia julibrissin*
CBT-i: Cognitive behavioral therapy for insomnia
OSA: Obstructive sleep apnea
TCM: Traditional Chinese medicine
OCT: 3-octanol
MCH: 4-methylcyclohexanol
PI: Performance index
CAM: Chinese alternative medicine.

## Supplementary Materials

Supplementary Materials The HPLC fingerprint for quality control of A. julibrissin.
