# Supplementary figures and images for: Albizia julibrissin Ameliorates Memory Loss Induced by Insomnia in Drosophila
Source: Evid Based Complement Alternat Med. 2019 Apr 1;2019:7395962. doi: 10.1155/2019/7395962 (PMC6463593; doi:10.1155/2019/7395962)

## Slide 1
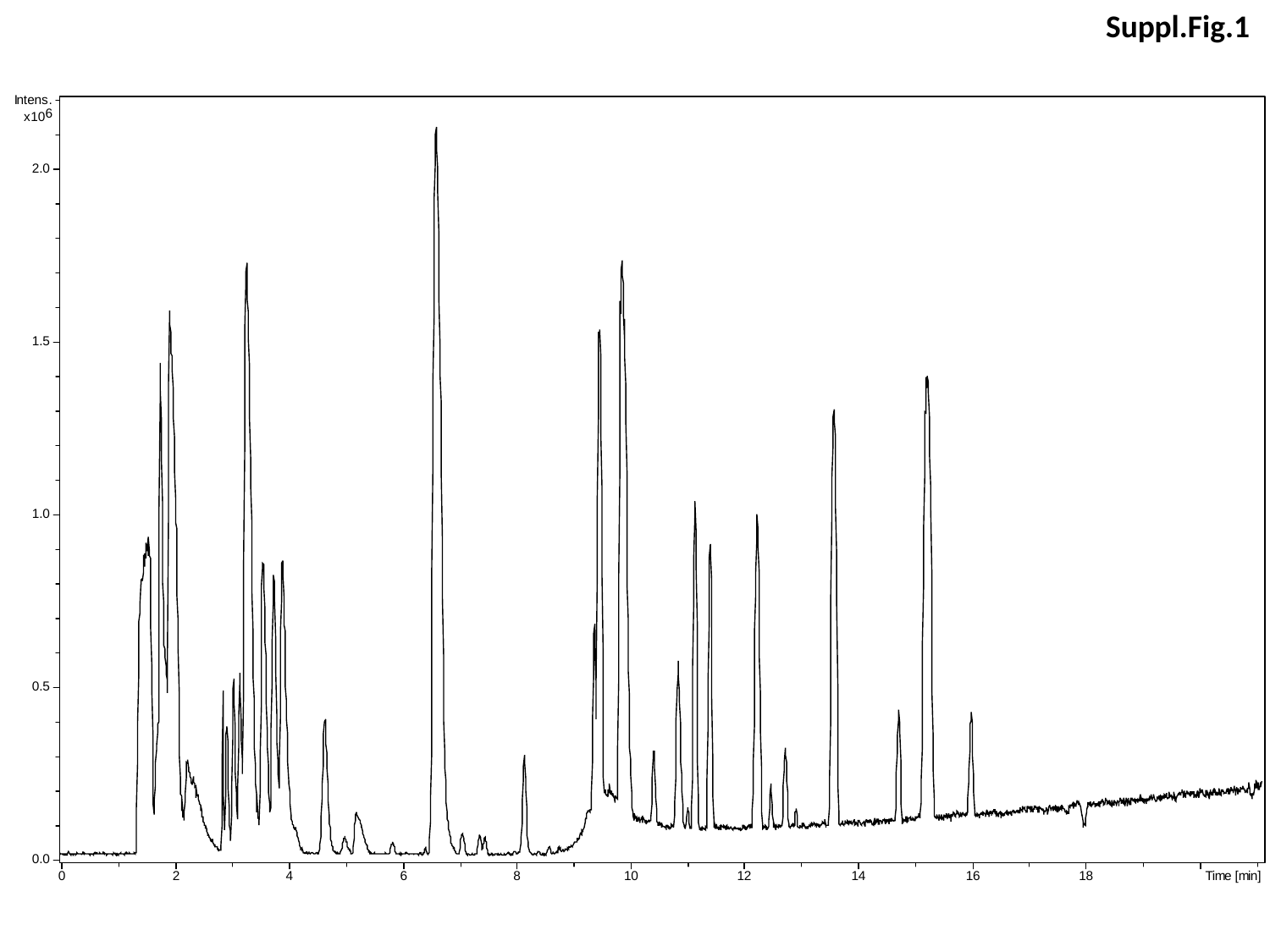

Suppl.Fig.1

Supplement: Supplementary Materials — The HPLC fingerprint for quality control of A. julibrissin. [file 7395962.f1.pptx]
